# Post-treatment Vascular Leakage and Inflammatory Responses around Brain Cysts in Porcine Neurocysticercosis

**DOI:** 10.1371/journal.pntd.0003577

**Published:** 2015-03-16

**Authors:** Siddhartha Mahanty, Miguel Angel Orrego, Holger Mayta, Miguel Marzal, Carla Cangalaya, Adriana Paredes, Eloy Gonzales-Gustavson, Gianfranco Arroyo, Armando E. Gonzalez, Cristina Guerra-Giraldez, Hector H. García, Theodore E. Nash, the Cysticercosis Working Group in Peru

**Affiliations:** 1 Laboratory of Parasitic Diseases, National Institutes of Allergy and Infectious Diseases, National Institutes of Health, Bethesda, Maryland, United States of America; 2 Laboratory of Immunopathology, Universidad Peruana Cayetano Heredia, Lima, Peru; 3 Facultad de Ciencias y Filosofía, Universidad Peruana Cayetano Heredia, Lima, Peru; 4 Faculty of Veterinary Sciences, Universidad Nacional Mayor de San Marcos, Lima, Peru; 5 Cysticercosis Unit, Instituto Nacional de Ciencias Neurologicas, Lima, Peru; University of Queensland, AUSTRALIA

## Abstract

Cysticidal treatment of neurocysticercosis, an infection of humans and pig brains with *Taenia solium*, results in an early inflammatory response directed to cysts causing seizures and focal neurological manifestations. Treatment-induced pericystic inflammation and its association with blood brain barrier (BBB) dysfunction, as determined by Evans blue (EB) extravasation, was studied in infected untreated and anthelmintic-treated pigs. We compared the magnitude and extent of the pericystic inflammation, presence of EB-stained capsules, the level of damage to the parasite, expression of genes for proinflammatory and regulatory cytokines, chemokines, and tissue remodeling by quantitative PCR assays between treated and untreated infected pigs and between EB-stained (blue) and non stained (clear) cysts. Inflammatory scores were higher in pericystic tissues from EB-stained cysts compared to clear cysts from untreated pigs and also from anthelmintic-treated pigs 48 hr and 120 hr after treatment. The degree of inflammation correlated with the severity of cyst wall damage and both increased significantly at 120 hours. Expression levels of the proinflammatory genes for IL-6, IFN-γ, TNF-α were higher in EB-stained cysts compared to clear cysts and unaffected brain tissues, and were generally highest at 120 hr. Additionally, expression of some markers of immunoregulatory activity (IL-10, IL-2Rα) were decreased in EB-stained capsules. An increase in other markers for regulatory T cells (CTLA4, FoxP3) was found, as well as significant increases in expression of two metalloproteases, MMP1 and MMP2 at 48 hr and 120 hr post-treatment. We conclude that the increase in severity of the inflammation caused by treatment is accompanied by both a proinflammatory and a complex regulatory response, largely limited to pericystic tissues with compromised vascular integrity. Because treatment induced inflammation occurs in porcine NCC similar to that in human cases, this model can be used to investigate mechanisms involved in host damaging inflammatory responses and agents or modalities that may control damaging post treatment inflammation.

## Author Summary

Neurocysticercosis is caused by infection of the brain with the larval (cyst) stage of the tape worm *Taenia solium* in humans and pigs. Antiparasitic drug treatment is compromised by worsening of neurological symptoms during therapy due to reactive inflammation triggered by the dying parasite. The immune mechanisms that cause this inflammation are poorly understood. In this study, we investigated the nature of inflammation after treatment in pigs naturally infected with *T*. *solium* cysts. Evans blue dye injected into infected pigs marks areas in the brain where the normally impermeable capillaries have become more permeable, allowing damaging cells and molecules to leak out into the brain. By microscopy and measurement of gene expression for inflammation-inducing immune mediators, we show that inflammation in the brain tissues around cysts is more severe with increased vessel leakage. Furthermore, the levels of these mediators increased after antiparasitic drug treatment. A significant implication of these findings is that it may be possible to inhibit the inflammation around parasites using drugs or biologics that inhibit these inflammatory pathways and, thereby, reduce local brain damage during treatment. These observations may also be applicable to other inflammatory conditions that affect the brain.

## Introduction

Neurocysticercosis (NCC), an infection with the larval stage of the tapeworm, *Taenia solium*, is the most common cause of adult onset epilepsy worldwide. Infection is acquired from adult tapeworm carriers who excrete infectious ova either within proglottids or free in the feces. Following ingestion of feces by free roaming pigs or accidental ingestion of ova by humans, the ova release oncospheres that burrow into the intestine, enter blood vessels and disseminate to the tissues, where they develop into viable larval cysts mostly in the muscles, subcutaneous tissue and brain. The life cycle is completed when tapeworms develop in the small intestine after humans ingest raw pork containing cysts. Symptomatic disease is the consequence of brain and spinal cord infection and its severity and course are determined by the location, number and degree of inflammation directed to the cysts [1,2].

Viable cysts invoke little host inflammatory response. For reasons that are unclear, degenerating cysts or those damaged due to anthelmintic treatment provoke pathologic host inflammatory responses [1–3]. Inflammation around degenerating cysts in the brain parenchyma usually give rise to seizures while those in the subarachnoid spaces cause diffuse and/or focal arachnoiditis resulting in hydrocephalus, infarcts and nerve entrapments. Cysts in the ventricles commonly cause hydrocephalus due to mechanical obstruction of CSF outflow or as a result of ventriculitis and scarring [2].

The detrimental inflammatory response induced by cysticidal drugs complicates treatment; in practice, corticosteroids are almost always employed to suppress inflammation and control symptoms. The optimal regimen for the safe and effective use of corticosteroids or other anti-inflammatory agents have not been studied in multicystic or complicated NCC. Therefore the dose, duration and type of corticosteroid selected are based upon the experience of the individual practitioner [4]. A better understanding of the acute inflammatory responses induced by treatment is necessary to devise simple, safe and more effective treatment measures.

The pig is the natural intermediate host of *T*. *solium* and the only other animal readily infected with cysts of *T*. *solium*. Although used intermittently over the years for a variety of studies [5–9], pigs have not been utilized for the development of an animal model for cysticercosis or neurocysticercosis. Since the pig harbors cysts in both the brain and muscles, it has the potential to serve as an excellent model of human infections, and provides opportunities to study the pathogenesis of inflammation as well as immunoregulation in the vicinity of live and dying cysts. A limited number of studies investigating the pathology and underlying mechanisms of inflammation around cysts in naturally infected pigs have shown that the host reaction to the cyst results in a progressive local inflammatory response that develops into a fibrotic reaction with collagen deposition and granuloma formation [5,8]. The granuloma formation is characterized by infiltration with mononuclear cells and eosinophils [5,6,8,10], accompanied by apoptosis of infiltrating lymphocytes in early degenerating cysts and neuronal degeneration as well as astrogliosis [11,12], in the brain tissue adjacent to granulomas [11]. A comparison of histopathology with magnetic resonance imaging (MRI) confirmed a correlation of MRI findings with viable, early and late degenerating cysts [13,14].

Extravasation of vascular fluids around cysts, often revealed by contrast enhancement and/or edema in radiological imaging (computed tomography [CT] or MRI), appears to be a crucial element of the pathology around degenerating cysts and a feature of post-treatment pericystic inflammation [10,15]. Using extravasation of intravenously delivered Evans blue (EB) as a marker for vascular leakage in and around cysts [16], we investigated the relationship between vascular permeability around cysts and the induction of inflammatory pathways following treatment with anthelmintic drugs. Gene expression analysis of the pericystic tissues of a subset of brain cysts revealed that increased permeability following anthelmintic treatment is accompanied by an upregulation of proinflammatory genes and a corresponding down regulation of some regulatory (anti-inflammatory) genes. These data reveal a close relationship between vascular leakage and pericystic inflammation in neurocysticercosis and confirm the utility of a pig “model” of infection to study inflammatory mechanisms involved in this disease.

## Materials and Methods

### Study animals

Thirteen outbred pigs naturally infected with *Taenia solium*, as determined by a positive tongue examination, were obtained in Huancayo, Peru [17]. Pigs were anesthetized with ketamine (10 mg/K intramuscular injection, Agrovetmarket SA, Peru) and xylazine (2 mg/Kg, Agrovetmarket SA, Peru), for intravascular infusion and euthanized with sodium pentobarbital (100–125 mg/Kg, intravenous injection, Agrovetmarket SA, Peru). The study protocol and procedures were reviewed and approved by the Ethics Committee of the Veterinary School of San Marcos University in Lima, Peru. Pigs received one of three treatments prior to EB infusions: 1) no anthelmintic treatment (infected, n = 5); 2) praziquantel (100 mg/kg po, once) two days earlier (infected, n = 4,) or, 3) praziquantel (100 mg/kg po, once) five days earlier (infected, n = 4).

### Ethics statement

Animal experiments were performed at the Facultad de Medicina Veterinaria, Universidad Nacional Mayor de San Marcos, Lima, Peru, under the protocol “Evaluación de la permeabilidad vascular en cerebro y músculo de cerdos naturalmente infectados con *Taenia solium*”, with Dr. Armando Gonzalez as principal investigator. The study protocol was approved by the Animal Ethics and Wellbeing Committee of the University (Constancia de autorización ética No. 006, November 2010) and comply the National Institutes of Health/AALC guidelines.

### Treatment conditions and EB infusion

In two separate experiments infected pigs were treated with a single dose of praziquantel (100 mg/Kg, Merck, Darmstadt, Germany) and sacrificed 48h (n = 4) and 120h later (n = 4). Five untreated infected pigs were used as controls. Two hours before euthanasia, pigs were anesthetized and infused with 2% Evans blue (EB, 80 mg/Kg; Sigma-Aldrich, St. Louis, MO) in normal saline (NaCl 0.85%, Laboratorios Baxter, Colombia, Baxter del Peru) by intravenous injection via the carotid artery. Just after euthanasia, the pigs were perfused with chilled saline solution with heparin (NaCl 0.85% with 10 UI of heparin/mL) and the brain was immediately removed at necropsy.

### Collection and selection of specimens for qPCR and histology

Brains were cut into ~1 cm slices on dry ice. The presence of blue and clear stained tissue around cysts was documented by gross examination. Of a total of 371 cysts harvested for this study, 30 (8%) of capsules were randomly selected for qPCR analysis. The samples selected, including cysts with both blue and clear pericystic capsular tissues, were harvested and placed in chaotropic buffer (RNALater, Qiagen/Life Technologies, Gaithersburg, MD) for further processing. For histopathological studies, the remainder of the brain cysts including the pericapsular tissue were fixed in 10% neutral buffered formalin. Samples from unaffected brain parenchymal tissue clearly distant from pericystic tissues were used as reference tissues for qPCR analysis.

### Histological examination

Paraffin sections were processed using standard procedures and stained with hematoxylin and eosin (HE) and Masson’s Trichrome stain (MT). Only complete cysts (151/371, or 41%) were used because the generation of the inflammatory and cyst damage scores required inclusion of the circumference. Quantitative inflammatory responses were assessed histologically in cysts with complete capsules and/or cyst walls in bright field microscopic images (Primo Start, Zeiss, Germany) of each cyst using a calibrated camera (AxioCam ICc1, Zeiss, Germany) with AxioVision v4.6 software (Zeiss, Germany).

Initially, an image of each complete cyst including the surrounding host reaction (capsule) was examined to determine the range of inflammatory changes present around each cyst and to calculate the proportion of the cyst circumference showing each of the designated inflammatory scores (IS) 0 to IS 4 (see S1 Table and S1 Fig.). The classification of the inflammatory stages broadly followed the schema described by Álvarez *et al* [5] and Londoño *et al* [8] with additional semiquantitative assessment of the extent and severity of the inflammation around each cyst, based on the average number of cells per high power field and the area of the pericystic tissue that contained the inflammatory infiltrates (See S1 Fig.). Using these measurements, IS 1 to 4 represented increasing extent and severity of inflammatory reactions (S1 Fig.). The presence of typical granules and nuclear morphology allowed us to identify eosinophils on HE stains, but for determination of semi-quantitative inflammatory and cyst wall damage scores we modified the approach used by Alvarez *et al*. [5], using an assessment of the location and density of total cellular infiltrate without identifying constituent cell types.

Cyst wall damage was categorized into five stages of damage corresponding to damage scores (DS)0 to DS4, based on the degree of tissue disruption in the cyst walls, as outlined by Londoño *et al*. [8], defined in S1 Table and illustrated in S1 Fig.

The IS and DS scores were combined with the percentage of capsule around each cyst with each stage of inflammation or damage were used to generate composite inflammatory (IS-composite) and damage scores (DS-composite), defined for each cyst using the formula: IS composite = Sum (IS Score x % of capsular circumference) DS-composite = Sum (DS Score x % of cyst circumference) For the purpose of this calculation, the percentage of the circumference was rounded off to increments of 20% (i.e., 0, 20, 40, 60, 80 or 100%). As an example, a cyst-capsule that had 20% of the capsule with IS 2, 40% with IS 3 and 40% with IS 4 would have the composite IS score of 320 (2x20+3x40+4x40 = 320). Similar calculations were used for composite DS scores. Using these formulae the theoretical maximum value for the IS- and DS-composite scores for a given cyst is 400 (4 x 100%).

### Generation of cDNA and real time PCR (qPCR) assays for gene expression studies

Total RNA was isolated from 50–100 mg of tissue samples using TRIzol reagent (Invitrogen, Carlsbad, CA). RNA concentrations were determined using a NanoDrop (NanoDrop Products, Wilmington, DE). cDNA was generated from one microgram of extracted total RNA using multiscribe RT polymerase (Applied Biosystems, Inc, Bedford, MA) in 100-ul reactions by incubation for 10 min at 25°C followed by 60 min at 37°C, 5 min at 95°C on a thermocycler (MJ Research PTRC-200, BioRad-MJ Research, Hercules, CA). Real-time PCR was performed in 20 μl reactions using TaqMan Gene Expression Assays (Applied Biosystems, Foster City, CA) and primer probe pairs for each gene of interest. PCR reactions, run in triplicate, used the following cycling parameters: 40 cycles of 20 sec at 95°C, 1 sec at 95°C and 1 min at 60°C, on an AB StepOnePlus cycler, normalized against 18S ribosomal RNA as a control gene. Primers and probes used were off-the-shelf reagents purchased from the vendor (Life Technologies, Grand Island, NY). We used 18S rRNA (Life Technologies code 4319413E) as a control gene to confirm RNA integrity and primer probe pairs for porcine IL-1β (Ss03393804_m1), IL-6 (Ss03384604_u1), IL-13 (Ss03392353_m1), IFN-γ (Ss03391053_g1), TNF-α (Ss03391318_g1), IL-10 (Ss03391318_g1), CTLA4 (Ss03213761_m1), IL-2RA (CD25; Ss03381754_u1), FoxP3 (Ss03376695_u1), matrix metalloprotease (MMP) 1 (Ss03374796_u1), MMP9 (Ss03392097_g1), tissue inhibitor of metalloprotease (TIMP) 1 (Ss03381944_u1) and TIMP2 (Ss03375440_u1) genes. PCR reactions, run in triplicate, used the following cycling parameters: 40 cycles of 20 sec at 95°C, 1 sec at 95°C and 1 min at 60°C, on an AB StepPlusOne cycler (Life Technologies, Grand Island, NY). Gene expression was analyzed as anti-log2 transformed threshold cycle number corrected for the housekeeping gene (2-^ΔΔCT^), representing a fold increase (or decrease) over the control gene.

### Statistical analysis

Non-parametric statistics, Mann-Whitney U test for two groups and Kruskal-Wallis test for multiple groups, were calculated using Prism software (Graphpad, San Diego, CA) for comparisons of the histological and gene expression parameters mentioned above between uninfected and infected pigs and between clear capsules and those with EB staining. Differences with p-values of <0.05 were considered statistically significant.

## Results

### Study population and treatment subgroups

We studied brain tissue samples from thirteen infected pigs for experimental data. Examination of the brain immediately post mortem revealed numerous clear and blue-stained capsules in situ on the surface of the brain, and abundant blue stained capsules throughout the muscles. The unrestricted extravasation of EB in muscles has been noted previously [16], limiting the usefulness of EB extravasation to studies of the brain. A total of 151 capsules from the brains of 11 pigs were examined histopathologically (using haematoxylin-eosin staining) for evidence of inflammation and damage to the cyst wall. An available sample set of thirty capsules (20 blue and 10 clear) from the brains of 6 pigs were selected randomly among cysts with complete capsules for assessment of gene expression of pro-inflammatory and regulatory pathway genes by qPCR. Different capsule samples were used for gene expression and for histopathological analyses.

### Assessment of Evans Blue extravasation

**Fig 1:**
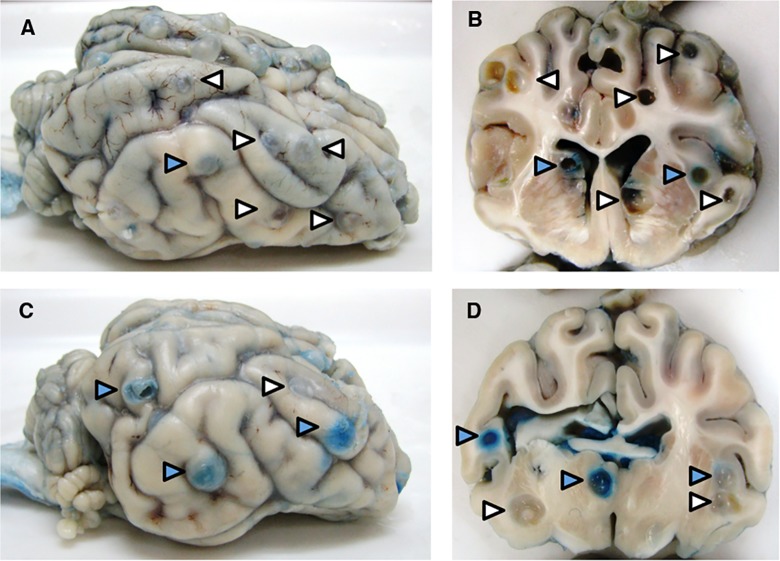
Clear and blue-stained capsules (EB capsules) on the surface and cut surfaces of brains from infected pigs. Photographs of the surface and coronal sections of representative brains from an untreated pig (A and B) and a pig treated with praziquantel 48 h previously (C and D), showing cysts with clear (white arrowheads) and blue capsules (blue arrowheads) visible within the parenchyma and on the surface.

The presence and number of both cysts with clear and blue capsules was confirmed in 1-cm coronal sections of both cerebral hemispheres in all pigs that received EB infusions (Fig. 1) as previously reported [16]. The proportion of cysts with capsules that demonstrated EB staining was significantly higher in PZQ treated than in untreated infected pigs [16]. The increase in EB stained capsules is consistent with the increase in enhancement reported in treated patients within the first week after the start of cysticidal agents [2,4]. Among these 371 total cyst samples, 33 of 47 (70%) cysts from untreated and 89 of 104 (86%) cysts from treated infected pigs demonstrated EB extravasation.

### Relationship between inflammation, cyst damage and Evans Blue extravasation

**Fig 2:**
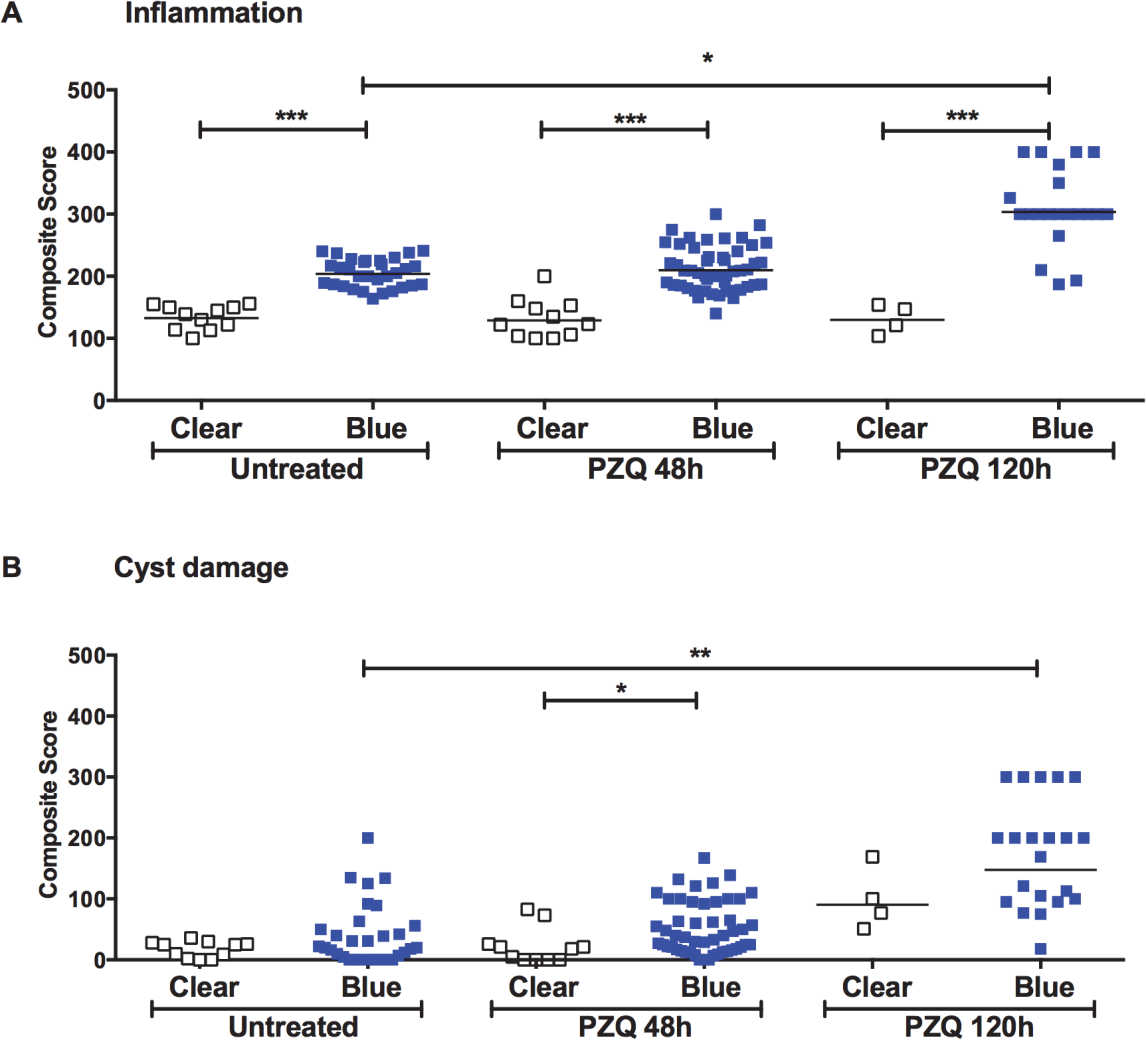
EB capsules show increased inflammation and cyst wall damage compared to clear capsules. Cysts were harvested from the brains of infected untreated pigs (n = 3; see Methods) or pigs treated with PZQ 48h (n = 4) or 120h (n = 4) earlier. For each cyst, scores reflecting the degree and extent of the inflammatory cell infiltrates (A) and cyst wall damage (B) at 48h (PZQ 48h) and 120h (PZQ 120h) after PZQ treatment (see Methods and S1 Fig.) are shown (open squares refer to clear capsules and closed squares, to EB capsules, bar = mean). Statistical significance in comparison between groups is represented by asterisks (*: p<0.05; **: p<0.01; ***: p<0.005).

Semiquantitative histologic inflammatory and cyst damage scores were determined in 151 cysts with an evaluable capsule and cyst. Irrespective of anthelmintic treatment (i.e., in all pigs, untreated, and at 48 hr and 120 hr post-treatment), blue capsules had significantly higher inflammatory scores compared to clear capsules (Fig. 2). In addition, the inflammatory scores of blue capsules increased between 48 hr and 120 hr of treatment but the difference in inflammatory or damage scores between clear and blue capsules only reached significance at 120 hr. In contrast to capsules with EB staining, no significant changes in either inflammation or damage were found between clear capsules in treated pigs at all time points.

We analyzed cyst damage scores for both the effect of treatment with PZQ and the effect of BBB disruption (EB stained capsules). We found that PZQ treatment alone did not significantly increase damage scores, and that increased scores were seen only in cysts with EB stained capsules in PZQ treated pigs not only when compared with untreated cysts but also when compared to treated clear cysts (Fig. 2B). In contrast, damage scores for clear capsules were not significantly increased in pigs treated with PZQ when compared to untreated pigs (Fig. 2B). As shown in Fig. 2B, damage scores of cysts with EB-stained capsules at 120 hr post-treatment were significantly increased above both the corresponding cysts with EB stained capsules at 48 hr as well as cysts with either EB-stained or clear capsules in untreated pigs (Fig. 2B). The increase in the proportion of EB capsules over time as well as the increase in inflammatory and cyst damage scores at 120 hr indicates that treatment had induced or exacerbated pericystic inflammation and cyst damage following praziquantel treatment, an expected result.

### Upregulation of pro-inflammatory and regulatory genes in cysts with Evans Blue leakage

**Fig 3:**
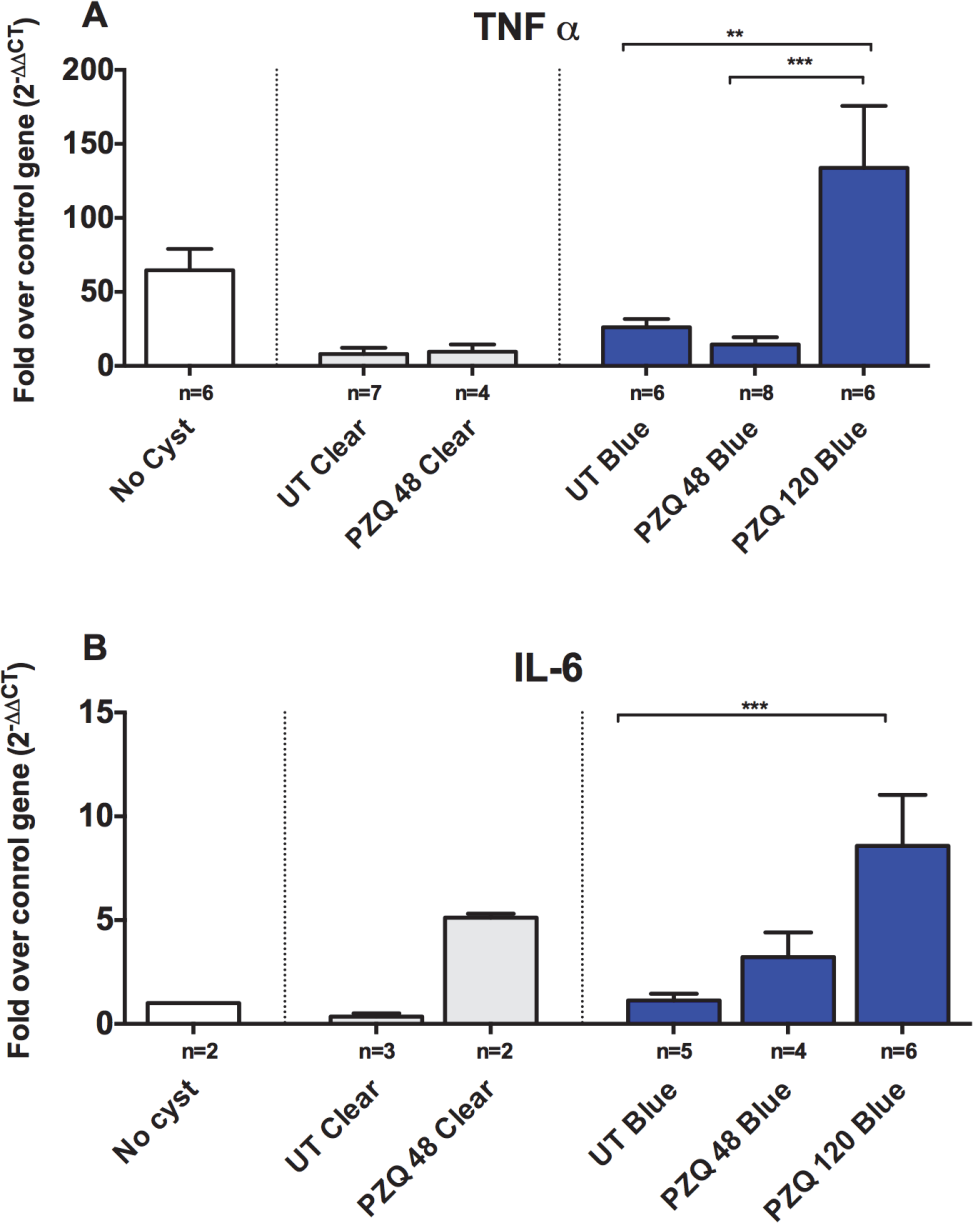
Upregulation of proinflammatory genes in EB capsules compared to clear capsules. Expression levels of RNA in six types of brain tissue samples, uninfected brain tissue distant from cysts (no cysts), clear pericystic brain tissue in untreated pigs (UT Clear) and at 48h post treatment (PZQ 48 Clear) and EB capsules in untreated pigs (UT Blue) and at 48h (PZQ 48 h Blue) and 120h post treatment (PZQ 120 h Blue) were quantified using real-time reverse transcriptase polymerase chain reaction (RT-PCR) assays. Gene expression levels are depicted as fold increase of studied RNA over reference (housekeeping) RNA (18S rRNA), TNF-α (A; number [n] of samples for the indicated types of capsules are shown below each bar) and IL-6 (B). The bars represent means and CI_95_ for the group. Statistically significant differences in levels of gene expression are indicated by asterisks (p<0.05, Mann-Whitney U test). Asterisks represent level of significance: *: p<0.05; **: p<0.01; ***: p<0.005.

**Fig 4:**
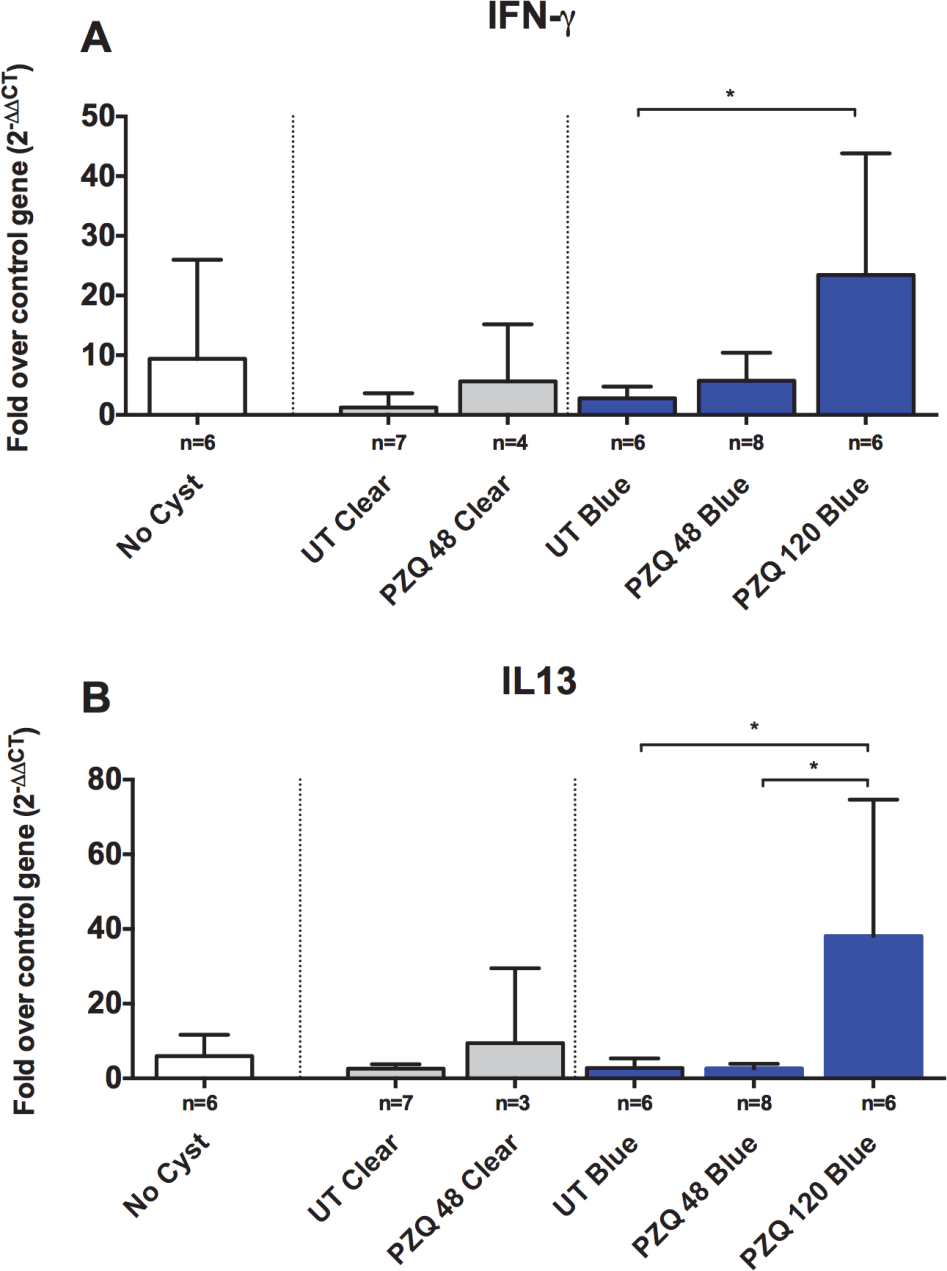
Upregulation of both Th1 and Th2 markers, IFN-γ and IL-13, in blue capsules following PZQ treatment. Expression levels of RNA in brain tissues and EB-stained and clear capsules for IFN-γ (A; number [n] of samples for the indicated types of capsules are shown below each bar), and IL-13 (B). In each graph, the bars represent means and CI_95_ for the group. Statistically significant differences in levels of gene expression are indicated by asterisks (p<0.05, Mann-Whitney U test). Asterisks represent level of significance: *: p<0.05; **: p<0.01; ***: p<0.005.

**Fig 5:**
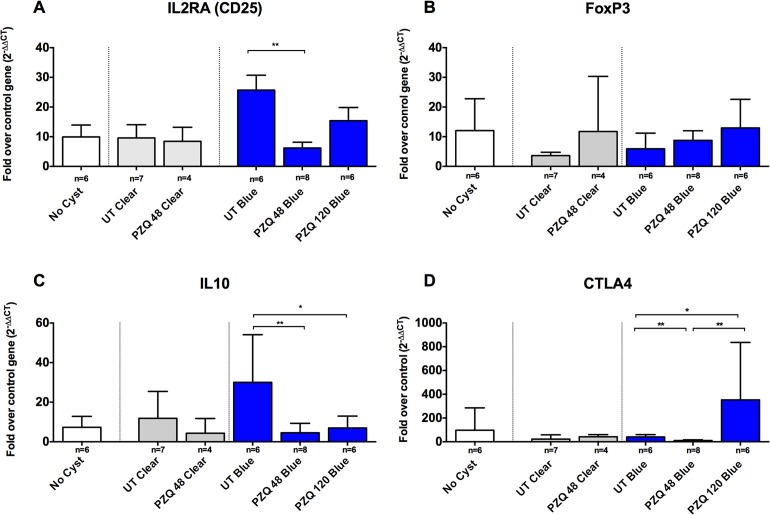
Increased expression of CD-25 and IL-10 in EB capsules following PZQ treatment. Expression levels of RNA in brain tissue samples from EB-stained and clear capsules were quantified (See Methods). Shown are expression levels, depicted as fold increase over the RNA levels for the reference (housekeeping) gene (18S rRNA), encoding four representative regulatory T cells (Treg) phenotyping and functional markers: CD25 (A; number [n] of samples for the indicated types of capsules are shown below each bar), FoxP3 (B), IL-10 (C) and CTLA4 (D). Statistically significant differences in levels of gene expression are indicated by asterisks (p<0.05, Mann-Whitney U test). Asterisks represent level of significance: *: p<0.05; **: p<0.01; ***: p<0.005.

Given that there was a significant increase in the inflammatory reaction around the blue cysts after PZQ treatment, we investigated a number of pro-inflammatory and immunoregulatory pathways to identify molecules involved in regulating these responses in vivo. As was observed with damage scores, clear capsules did not show significant changes in the levels of gene expression for inflammatory markers (Figs. 3–5) at 48h post treatment, however, it should be noted that no clear capsules were analyzed for gene expression analysis at 120h because all cysts harvested for RNA extraction were found to have EB stained capsules. In contrast, as shown in Figs. 3, 4 and 5, there was a relative increase in expression levels of all the proinflammatory gene tested (TNF-α, IL-6 and interferon (IFN)-γ) in EB-stained capsules compared to clear capsules from untreated pigs and brain tissues from infected pigs distant from the cyst location. Interestingly, IL-13, a gene associated with fibrosis and Th2 responses in other helminth infections, also increased in EB-stained capsules, but not in clear capsules, at 120 hr post treatment (Fig. 4). Differences between blue and clear capsule-associated cysts at 120 hr and at 48 hr did not achieve statistical significance, likely due to the small number of clear cysts available for analysis.

Unlike the proinflammatory markers, a variable pattern of change in gene expression was observed in the regulatory genes evaluated. IL-10 and CD25 decreased transiently after treatment by 48 hr post treatment in the EB capsules, whereas, CTLA4 and FoxP3 showed transiently reduced expression levels in EB-stained cysts at 48 hr post treatment, which rebounded to higher levels at 120 hr (Fig. 5). These data indicate that higher expression of proinflammatory genes is accompanied by a transient reduction in the levels of mRNA for several regulatory genes. However, all the significant changes were seen in EB-stained cysts; clear cysts did not demonstrate an increase in pro-inflammatory or regulatory genes (See Figs. 3–5). Of note, there were no clear capsules in the brains of animals studied for gene expression 120 hr post treatment with PZQ, because the few cysts with clear capsules were used for histopathologic analysis.

**Fig 6:**
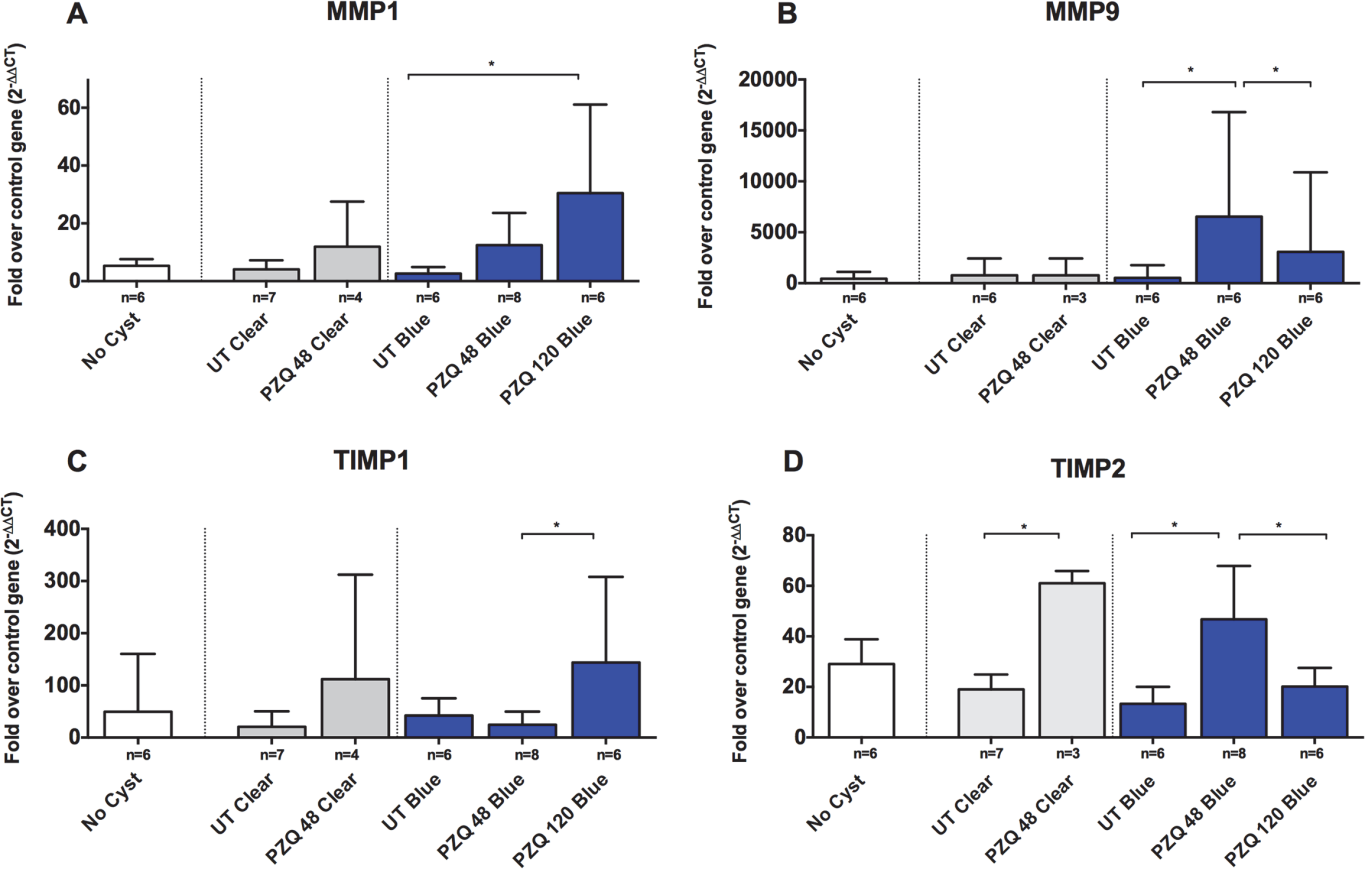
Increased expression of fibrosis/granuloma promoting genes in EB and clear capsules. Methods and tissues examined are identical to those analyzed in Figs. 3–5. Shown are four genes representative of fibrosis and granuloma promoting molecules: MMP1 (A; number [n] of samples for the indicated types of capsules are shown below each bar; n = 7, 6, 3, 6, 6, respectively), MMP9 (B), TIMP1 (C) and TIMP2 (D). Statistically significant differences in levels of gene expression are indicated by asterisks (p<0.05, Mann-Whitney U test). Asterisks represent level of significance: *: p<0.05; **: p<0.01; ***: p<0.005.

To determine if expression of genes associated with tissue remodeling and possibly in granuloma formation were increased, as reported in other animal models of NCC [18], we compared gene expression levels of matrix metalloproteases (MMP) 1 and 9 and tissue inhibitors of MMPs (TIMPs) 1 and 2 (Fig. 6) in capsules with and without EB staining. There were no significant differences in expression of these genes between EB-stained and clear capsules or uninfected tissues (Fig. 6). In contrast, expression of the genes for of MMP1 and MMP9, as well as the TIMP1 and TIMP2 increased significantly in EB-stained (blue) capsules at 48h and 120 hr after PZQ treatment. Interestingly, the increase in expression of MMP1 and MMP9 peaked at different times (48h for MMP9 and 120h for MMP1).

## Discussion

This pig model exhibits many of the features of the post cysticidal inflammatory reaction, which occurs in humans during the first week of antiparasitic treatment. In humans, the accompanying MRI changes include the development or exacerbation of gadolinium enhancement with or without surrounding edema. This response and its effect on the surrounding brain tissue give rise to treatment-induced seizures, which limit the usefulness of cysticidal treatment. The use of EB injection allowed anatomic identification and analysis of cysts with BBB leakage, which is equivalent to gadolinium extravasation around cysts during MRI. By performing semi-quantitative analysis of the infiltrating cyst-associated inflammatory cells, the expression of genes for cytokine and other regulatory molecules and the extent of cyst damage over time in relation to BBB leakage, we were able to determine the dynamics and relationship of treatment-induced changes to the pathology.

Our most significant finding was that treatment with praziquantel led to the induction of inflammation and blood-brain barrier disruption 48 hr and 120 hr post treatment similar to the increase in pericystic enhancement and perilesional edema observed by MRI in treated humans [19–21]. Using EB injection we found a positive correlation between the increase in inflammatory pathology and BBB disruption over time as shown by the increase in EB stained capsules. In comparison, cysts without BBB disruption, identified by a lack of EB staining, for the most part showed little inflammatory cell reaction or changes in cytokine gene expression. The cysts with acute inflammatory responses and BBB leakage and were associated with elevated expression of proinflammatory cytokines, but somewhat unexpectedly, also demonstrated heightened immunoregulatory responses, notably, of IL-13 and CD25 (IL-2 receptor). The correlation of the BBB leakage with inflammation is logical and expected since the pericystic inflammatory response primarily consists of white blood cells from outside the brain, which require enhanced access to the cyst by way of blood vessels located within the capsule.

In these experiments the inflammatory response directed to the treated, degenerating cysts and damage to the cysts apparently occurred at the same time. The magnitude of the inflammatory response was also found to be proportional to the degree of cyst wall damage. In addition, we found that the damage occurred predominantly in cysts that showed EB leakage in their capsules, best appreciated at 5 days post treatment (Fig. 2A). These findings are consistent with the notion that the inflammatory reaction and BBB disruption are likely important determinants of the degree of cyst damage. Our data are also consistent with previously reported inflammatory pathology in rodent models [22–25] and experimental pig infections [6,8–10]. In this study, we did not determine the types of inflammatory cells at the sites of EB leakage and cyst wall damage but focused on the intensity of inflammatory infiltrates by assessing the extent of infiltrate (described in S1 Table and depicted in S1 Fig.). However, eosinophils were notable and distinguishable by morphology and the presence of granules on HE staining (S1 Fig.).

The evidence for activation of inflammatory responses comes from increased expression of the pro-inflammatory mediators TNF-α, IL-6 and IFN-γ after treatment with PZQ (Figs 3 and 4A) and decreased levels of IL-10 in treated cysts (Fig. 5). However, the increased expression of inflammatory mediators (and conversely, lower levels of the regulatory molecule, IL-10), were seen around some, but not all, cyst types; the differences between untreated and PZQ-treated cysts were significant only in cysts that demonstrated BBB disruption (Figs. 4 and 5). These findings show that pre- and post-treatment degeneration of cysts and inflammation are closely tied to increased BBB disruption. The presence of gadolinium enhancement in human NCC is commonly used as a surrogate for the presence and degree of inflammation. These studies confirm the association.

An interesting observation was the post-treatment downregulation of IL-10, a major anti-inflammatory cytokine [9,18,19,26] and a marker of regulatory T cells (CD25) in the EB-stained capsules but not in unstained capsules (Fig. 5C). High levels of expression of mRNA for IL-10 have been reported in infected (but untreated) pigs previously [27]. Other markers indicative of modulation of inflammation, including FoxP3 (a marker for Treg cell populations) and CTLA4 (an inhibitory co-factor that functionally modulates Th and Treg cells and has been associated with T cell hyporesponsiveness in other chronic infectious diseases such as filarial infections [28] and in tuberculosis [29]), were upregulated in the same capsules that had lower levels of IL-10 and CD25 (Fig. 5). The high levels of IL-10 around untreated cysts or those without disruption of the BBB suggest that they are in a relatively hyporesponsive state and that treatment may be “releasing” or exposing the parasite to an unimpeded proinflammatory responses after the parasite is damaged or killed. In the proinflammatory environment, an increase in the expression of regulatory mediators, notably IL-13, may also represent a compensatory or homeostatic release of regulatory responses aimed at limiting potentially pathological inflammation.

Helminth infections are frequently associated with modulation of the host immune system [30]. The immunomodulatory state is a consequence of a dominant T helper (Th) type 2 adaptive response induced or associated with these infections, and is characterized by high levels of IL-4, IL-13, IL-5, IgE and IgG subtypes IgG1 and IgG4 specific for the parasite antigens [31], recruitment of immunomodulatory populations such as regulatory T cells (Tregs: CD4^+^CD25^+^FoxP3^+^ cells) [30,32] and inhibitory “alternatively activated” macrophages, or M2 macrophages [33,34]. Our results that show a relative lack of inflammation around untreated cysts (similar to unaffected, distant brain tissues) are consistent with an immunomodulatory state.

Although a regulatory environment appears to dominate in helminth infections [30,35] as well as in humans and pigs infected with *T*. *solium* [27,36] and in rodent models of NCC [25,34,37,38], a more inflammatory process with edema and inflammation around degenerating cysts has long been recognized as an important feature of NCC [2,39]. Symptomatic NCC is associated with higher levels of circulating pro-inflammatory mediators, including TNF-α, IFN-γ, IL-1β and IL-6 [40–43]. A good model of human infection should, ideally, reflect this mixed picture of inflammatory and regulatory immune activation. Indeed, this is what was observed in the cysts from pigs after PZQ treatment. The unexpectedly complex pattern of expression of different counterregulatory markers (IL-10 vs. CTL4 and FoxP3; Fig. 5) may be influenced by the anatomical location of the response. Since infection by this parasite occurs in the brain, in contrast to peripheral tissues, the immune response may differ from peripheral host responses, which are more reflective of naïve T and B cells, and monocytic cell populations than the tissue-derived immune cells found in the brain. The similarities in histopathology and evolution of the cysts in this pig model and the ability to study the parasite and host response directly in the brain make this a promising model for research in human NCC.

A number of molecules that play important roles in granuloma formation and fibrosis were also evaluated in the present study, since degenerating or dead cysts frequently demonstrate granulomatous inflammatory responses [6,8,44]. We focused on molecules that promote or inhibit tissue fibrosis, specifically, matrix metalloproteases (MMPs)1 and 9, and their inhibitors (tissue inhibitors of metalloproteases [TIMPs] 1 and 2) [45–48]. Proteins of the MMP family break down extracellular matrix in normal physiological as well as in pathological states, such as inflammatory reactions, arthritis and metastasis. TIMP1 and TIMP2 belong to the TIMP gene family, a family of MMP inhibitors that play important roles in regulation of fibrosis associated with MMPs [47–49]. Data from our experiments (Fig. 6) showed that capsules with impaired vascular integrity (EB-staining) had significantly upregulated expression of several of these proteins. Two patterns of expression were observed: MMP1 and TIMP1 increased progressively 48 to 120 hr after treatment whereas MMP9 and TIMP2 appeared to peak 48 hr post treatment with a subsequent reduction in levels of expression at 120 hr (Fig. 6). In this case, TIMP2 levels also increased in clear cysts 48 hr post treatment. These data suggest that tissue proteases likely play a role in tissue remodeling after treatment and are associated with inflammation around cysts damaged by PZQ treatment, a proportion of which may be destined to undergo fibrotic changes and calcification [50,51].

These in vivo studies are difficult, labor intensive and limited by the number of infected animals available. Changes earlier than 48 hr were not analyzed but may be important. In addition unlike MRI studies that can be performed sequentially and can demonstrate changes of the same cysts over time, in the present model the status of individual cysts before treatment cannot be determined. Instead, we show changes related to untreated animals and changes in pigs studied at earlier and later time points. In previous studies that focused on pathologic changes following anthelmintic treatment, we and others noted an increase in the eosinophilic migration into the pericystic region [9,10,52]. In a previous report we showed that eosinophils were at times numerous and associated with focal regions of cyst wall damage and infiltration, which increased with cysticidal treatment [10]. These regions were enriched in eosinophils, which had also ingested EB-bound albumin. The eosinophilic infiltrates may important if not essential for cyst degeneration but may also be responsible for much of the inflammatory side effects of anthelmintic treatment.

Although pigs, like humans, are natural hosts for *T*. *solium* and histopathological features and immune responses observed in pigs are similar to what has been surmised from human studies [19,39,53–55], there are important differences that advise caution in interpreting observations from the pig as a model for human disease. An obvious difference is the parasite burden, which is generally significantly higher in pigs than in humans [6,56]. The longevity of the infection is also shorter in pigs, and consequently there are no calcified cysts in the pigs used in our experiments. The heavy burden of extracerebral cysts in pigs is also at variance with human infections at the time of presentation. The heavy burden of parasite antigens that results may have a dampening effect on inflammatory responses, as has been seen in other helminth infections [31]. These factors have unknown influences on the nature of the immune response, and warrant further investigation in experimental infections with better defined infection parameters (such as parasite burden, duration of infection, etc).

In summary, our results indicate that BBB disruption is accompanied by both pro-inflammatory and regulatory host responses, and that the percentage of cysts with these characteristics increases with treatment. We have demonstrated BBB leakage directly by using EB, which allowed us to perform characterization of specific cysts presumably at different stages of damage or degeneration. We also found evidence of increased fibrosis in the pericystic capsular regions at both gene expression and histopathological levels in comparison to cysts that did not show BBB leakage. This model has many of the characteristics and features of human infections and therefore can be used to investigate the mechanisms involved in the genesis of inflammation, treatment-induced immunopathology and host parasite interactions. This will assist in identifying therapeutic agents with a narrower but more rational spectrum of targets to suppress inflammation in the CNS. Further studies in this model, using natural and experimental infections can be used for selection of better drugs and biological agents to suppress damaging post treatment inflammatory responses.

## Supporting Information

S1 Table Histological criteria used for staging of pericystic inflammatory reaction and cyst wall damage. Histological features of cysts capsules and cysts that define inflammatory stages (IS) 1–4 and cyst wall damage scores (DS) 1–4, respectively.
(DOCX)

S1 Fig Histological micrographs demonstrating the scale used to score inflammatory responses and cyst wall damage, as described in S1 Table. Panels A-D: Representative photomicrographs of cyst walls and pericystic tissues typical of inflammatory stage (IS)1 (A), IS2 (B), IS3 (C) and IS4 (D). Panels E-H: Representative photomicrographs of cyst walls illustrating damage scores (DS): DS0 (E), DS1 (F), DS2 (G) and DS3 (H). All tissue sections were stained with Masson´s trichromic stain. Magnification 400x in all panels. Key: collagen (co), cyst wall (cw), cellular infiltrate (ci), eosinophils (eos), tegument (t, hollow arrowhead), subtegument (st, gray arrowhead) and internal region (ir, black arrow head).
(TIF)
