# Supplementary material for: Post-treatment Vascular Leakage and Inflammatory Responses around Brain Cysts in Porcine Neurocysticercosis
Source: PLoS Negl Trop Dis. 2015 Mar 16;9(3):e0003577. doi: 10.1371/journal.pntd.0003577 (PMC4361613; doi:10.1371/journal.pntd.0003577)
Supplement: S1 Table — Histological features of cysts capsules and cysts that define inflammatory stages (IS) 1–4 and cyst wall damage scores (DS) 1–4, respectively. (DOCX) [file pntd.0003577.s001.docx]

**Supplementary Table 1: Histological criteria used for staging of pericystic inflammatory reaction and cyst wall damage**

| **Stage** | **Histopathologic features** |
| --- | --- |
| Inflammatory stage (IS) | |
| IS1 | Host inflammatory response absent or minimal with a thin collagen layer adjacent to cyst wall. Very few or no inflammatory cells. |
| IS2 | Moderate perivascular inflammatory cell infiltrate intermingled with abundant collagen fibers |
| IS3 | Abundant inflammatory cells interspersed with collagen; an epithelioid cell or eosinophil-rich layer close to cyst walls. |
| IS4 | Abundant inflammatory cells near and inside the cyst wall, with clear damage of cyst wall structures. Giant multinucleated cells close to the cyst. |
| Cyst wall damage score (DS) | |
| DS0 | No discernable alterations or disruption of tegument or other cyst structure |
| DS1 | Minimal alteration of cyst wall structures or tegument, but intact subtegumentary collagen layers and preservation of the cyst architecture |
| DS2 | Moderate alteration of cyst wall with indistinct tegumentary surfaces and disruption of subtegumentary layers. No changes in collagen layers below the subtegumentary layer |
| DS3 | Significant alteration of cyst wall with disruption or loss of microtriches on the surface of the tegument, disruption and vacuolation of the subtegumentary and internal collagen layers |
| DS4 | Severe alterations of cyst wall with loss of microtriches, and disruption or loss of subtegumentary layer with extensive vacuolation; loss of definition in the internal region of the cyst |

Adapted from Refs. 6. and 7
