# Supplementary figures and images for: Post-treatment Vascular Leakage and Inflammatory Responses around Brain Cysts in Porcine Neurocysticercosis
Source: PLoS Negl Trop Dis. 2015 Mar 16;9(3):e0003577. doi: 10.1371/journal.pntd.0003577 (PMC4361613; doi:10.1371/journal.pntd.0003577)

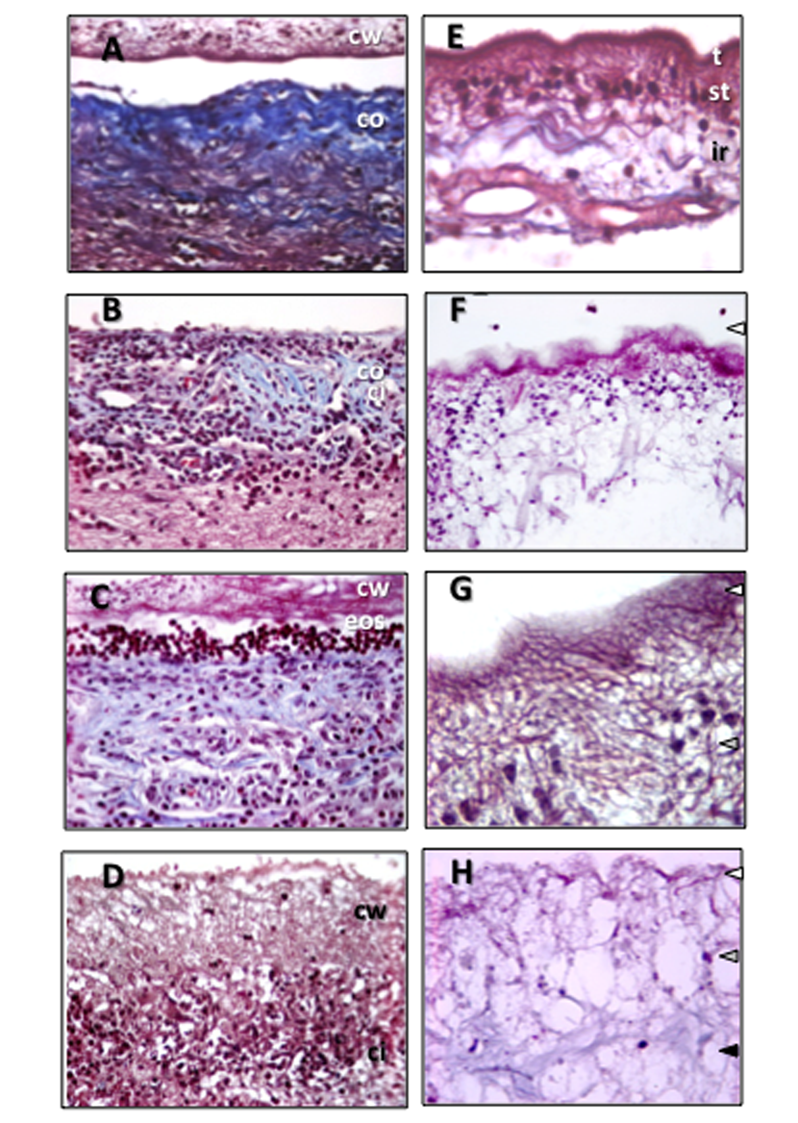

Supplement: S1 Fig — Panels A-D: Representative photomicrographs of cyst walls and pericystic tissues typical of inflammatory stage (IS)1 (A), IS2 (B), IS3 (C) and IS4 (D). Panels E-H: Representative photomicrographs of cyst walls illustrating damage scores (DS): DS0 (E), DS1 (F), DS2 (G) and DS3 (H). All tissue sections were stained with Masson´s trichromic stain. Magnification 400x in all panels. Key: collagen (co), cyst wall (cw), cellular infiltrate (ci), eosinophils (eos), tegument (t, hollow arrowhead), subtegument (st, gray arrowhead) and internal region (ir, black arrow head). (TIF) [file pntd.0003577.s002.tif]
